# Transcriptome Profiling and Differential Gene Expression in Canine Microdissected Anagen and Telogen Hair Follicles and Interfollicular Epidermis

**DOI:** 10.3390/genes11080884

**Published:** 2020-08-04

**Authors:** Dominique J. Wiener, Kátia R. Groch, Magdalena A.T. Brunner, Tosso Leeb, Vidhya Jagannathan, Monika M. Welle

**Affiliations:** 1 Department of Veterinary Pathobiology, College of Veterinary Medicine & Biomedical Science, Texas A&M University, College Station, TX 77843, USA; kgroch@cvm.tamu.edu; 2 Institute of Genetics, Vetsuisse Faculty, University of Bern, 3012 Bern, Switzerland; maggali.b@googlemail.com (M.A.T.B.); tosso.leeb@vetsuisse.unibe.ch (T.L.); vidhya.jagannathan@vetsuisse.unibe.ch (V.J.); 3 Dermfocus, Vetsuisse Faculty, University Hospital of Bern, 3010 Bern, Switzerland; monika.welle@vetsuisse.unibe.ch; 4 Institute of Animal Pathology, Vetsuisse Faculty, University of Bern, 3012 Bern, Switzerland

**Keywords:** dog, *Canis lupus familiaris*, dermatology, hair cycle, microdissection, transcriptome analysis, RNA-seq

## Abstract

The transcriptome profile and differential gene expression in telogen and late anagen microdissected hair follicles and the interfollicular epidermis of healthy dogs was investigated by using RNAseq. The genes with the highest expression levels in each group were identified and genes known from studies in other species to be associated with structure and function of hair follicles and epidermis were evaluated. Transcriptome profiling revealed that late anagen follicles expressed mainly keratins and telogen follicles expressed *GSN* and *KRT15*. The interfollicular epidermis expressed predominately genes encoding for proteins associated with differentiation. All sample groups express genes encoding for proteins involved in cellular growth and signal transduction. The expression pattern of skin-associated genes in dogs is similar to humans. Differences in expression compared to mice and humans include *BMP2* expression mainly in telogen and high *KRT17* expression in the interfollicular epidermis of dogs. Our data provide the basis for the investigation of the structure and function of canine skin or skin disease and support the use of dogs as a model for human cutaneous disease by assigning gene expression to specific tissue states.

## 1. Introduction

The skin is the largest organ, with various essential functions, including protection/barrier against outside influences and thermoregulation. The epidermis and its appendages form the outer layer of the skin and are the first shield of the body from external harm [1]. To fulfill the protective functions, an intact epidermis and appropriate numbers of hair follicles (HF) are necessary.

In all species, the interfollicular epidermis (IFE) is composed of a squamous, stratified, multilayered epithelium that frequently self-renews, involving epidermal stem and progenitor cells that reside in the basal layer of the epidermis [2,3]. After the basal epidermal cells detach from the basement membrane, their proliferation ceases and they undergo a terminal cell differentiation program, during which keratinocytes move upwards until they reach the cornified layer as flattened denucleated keratinocytes and are finally shed as squames at the skin surface [4,5,6].

The disturbance of this process can have a devastating impact, such as excessive water loss, invasion of pathogens and development of cancer, which may become life-threatening. The main role of HFs is the production of hair shafts to maintain the hair essential for a variety of functions, such as sensation and the dispersion of sebum and pheromones. In animals, the fur also has life-preserving functions, such as thermal regulation, visual communication and protection from solar radiation and physical trauma.

In contrast to the continuous renewal of the IFE, HFs renew periodically, going through life-long recurrent phases of growth (anagen), regression (catagen) and rest (telogen) [7,8]. The hair shaft is produced during anagen. The renewal of the IFE and the HF relies on stem cells and their progenitors. Several stem cell markers, such as LGR5, CD34, SOX9 and KRT15 (HF bulge stem cells markers) [9,10,11,12,13] or LGR-6 (expressed in the upper HF, sebaceous gland and IFE) have been described in humans and rodents [14,15] and their expression has also been investigated in dogs [10,16,17,18]. Stem cell quiescence and activation, as well as the subsequent events leading to epidermal differentiation and the maintenance of the hair cycle, are tightly regulated and involve numerous signals derived from epithelial, neuroendocrine and mesenchymal cells [19,20,21]. The reciprocal communication between signals from the immediate stem cell microenvironment within the IFE or the HF and signals from the larger macroenvironment are assembled in the stem cells. The interplay of proteins and all transcription factors implicated in several signaling pathways finally result in stem cell activation or quiescence. Once activated, stem cells give rise to rapidly proliferating transient amplifying cells, which terminally differentiate into the epidermal and the different hair follicle layers and the layers of the hair shaft. Important signaling pathways that regulate stem cell quiescence, proliferation and differentiation include the wingless-type mouse mammary tumor virus integration site (WNT)/β-catenin, transforming growth factor (TGF)-β, fibroblast growth factor (FGF)5, bone morphogenic protein (BMP), FGF18 and Notch signaling (reviewed in [22,23]). In general, BMP signals derived from dermal, adipose and epithelial tissue repress cell proliferation, while WNT and other signals promote stem cell activation and growth.

The maintenance of the protective function of the skin is not only dependent on a functional stem cell compartment but also on proteins involved in the structural and functional integrity. Examples are keratin intermediate filaments and proteins involved in cell–cell adhesion such as adherens junctions and desmosomes [24,25].

The immune function of the epidermis is maintained by the presence of specialized antigen presenting cells (Langerhans cells) and of numerous pattern recognition receptors, such as Toll-like receptors [26,27]. HFs harbor, for example, Langerhans cells and CD4+ or CD8+ T cells and produce chemokines and cytokines important in immune defense [28,29].

To date, many molecules involved in skin homeostasis have been extensively studied and their role in disease development in humans and rodents is well known [30,31,32]. Additionally, in domestic animals, numerous gene variants involved in genodermatoses were identified by whole genome sequencing and other approaches [33]. However, for some genes associated with skin disease, the function and anatomic location of the expressed proteins are unknown. Examples for this are *SUV39H2* variants in nasal parakeratosis or *FAM83G* variants in hereditary footpad hyperkeratosis [34,35].

Gene expression analysis is a useful tool to study the molecular mechanisms underlying skin development and homeostasis. The identification of differentially expressed genes (DEGs) in numerous disease phenotypes of humans and animals is increasingly applied in research.

The transcriptome sequencing of skin biopsies in dogs to identify disease phenotypes revealed numerous deregulated genes, some of which are well known and whose expression can thus be associated with a specific location. However, many of the expressed genes have neither been associated with disease nor attributed to a specific tissue or cell type [17,36]. Studies based on the transcriptome profiling of murine epidermis and HFs [37] and human skin [38,39,40] are available. To the best of our knowledge, for domestic animals, transcriptome data for specific anatomic locations of healthy skin are only available from plugged hairs or cell cultures derived from microdissected HFs of cashmere goats [41,42,43,44]. Although dogs are increasingly recognized as an attractive model for human disease, no whole transcriptome expression data of microdissected skin tissue have been published [45,46].

The aim of our study was to identify global gene expression and differential gene expression in microdissected canine HFs in late anagen and telogen and in the IFE using transcriptome analysis by sequencing of mRNA (RNA-seq). Furthermore, we performed ontological molecular analyses for DEGs in telogen and late anagen HFs. Our data provide the basis for the investigation of the structure and function of the canine skin or canine skin disease and for the use of dogs as a model for human cutaneous disease, by assigning the expression of different genes to specific tissue types.

## 2. Materials and Methods

### 2.1. Ethics Statement

All skin samples were taken from dogs that were euthanized for reasons unrelated to this study (such as internal disease or accident). All dogs were privately owned pets that were euthanized according to ethical standards of Texas A&M University, College Station, USA. The owners signed a consent form to donate their dead animal.

### 2.2. Skin Biopsies

Skin samples measuring approx. 2 × 2 cm were taken from the trunk from eight freshly euthanized dogs (dead for no longer than 12 h) of various breeds and age with no skin abnormalities (Supplementary Table S1). Most of the samples were derived from female spayed dogs.

### 2.3. Microdissection

From the skin samples of each dog, 20 single HFs were microdissected as described before by cutting above the sebaceous glands to separate HFs from IFE and, subsequently, the sebaceous glands were removed (Supplementary Figure S1) [47,48]. The follicular stages in each sample were assigned by the length of the hair shaft and the depth of the follicle within the tissue (according to the canine hair cycle guide by Müntener et al. [49]). All anagen HFs collected were in the anagen VI stage, identified by the hair bulbs located deep in the subcutis and the hair shafts emerging through the HF ostia [49]. While, in most dogs, either anagen or telogen follicles could be identified, in both dogs 4 and 8, late anagen and telogen hair cycle stages were visible. In these dogs, 20 single HFs in telogen and 20 HFs in late anagen were collected.

The IFE was microdissected by cutting it off from the dermis and the HFs were removed with a scalpel blade. Between 20 and 30 small IFE pieces of 1–3 mm were collected. Microdissected HFs and IFE pieces were stored in RNA*later* at −20 °C until RNA extraction.

### 2.4. Histological Analysis

Whole skin, adjacent to skin used for microdissection, was collected and fixed immediately in 10% buffered formalin. The formalin-fixed tissue was embedded in paraffin, cut as a 4 µm section and stained with hematoxylin and eosin (H&E) using standard procedures. The histological specimens were evaluated to confirm that no pathologic changes were present and to confirm the hair cycle stages determined by microdissection.

### 2.5. RNA Extraction and Transcriptome Sequencing (RNA-seq)

RNA for transcriptome sequencing was extracted from the 20 pooled microdissected HF of the same cycle stage and the IFE collected from each dog. RNA extraction, library preparation and RNA-seq were performed as described in a previous study [17,36]. In brief, total RNA was extracted using the RNeasy Micro Kit (Qiagen, Hombrechtikon, Switzerland) including proteinase K digestion according to the manufacturer’s protocol. Total RNA content was measured using the Qubit^®^ 2.0 Fluorometer (Invitrogen, Thermo Fisher, Basel, Switzerland) and RNA quality was assessed with a Bioanalyzer (Agilent 2100; Agilent Technologies, Basel, Switzerland). High quality RNA (RIN > 9) was reverse transcribed into cDNA with the SMART-Seq v4 Ultra Low Input RNA Kit (Takara, Saint-Germain-en-Laye, France) and libraries were constructed following the TruSeq^®^ Nano DNA Library Prep (Illumina, San Diego, CA, USA) protocol for RNA sequencing. Multiplexed total cDNA libraries were sequenced on two lanes using an Illumina HiSq3000 instrument with 100 bp single-end sequencing cycles. On average, thirty million reads were collected, converted into FASTQ file format and demultiplexed.

The data are available from the European Nucleotide Archive (ENA), study accession number PRJEB21761 and sample accessions SAMEA7016531-SAMEA7016551 [50].

### 2.6. Mapping to Reference Genome and Gene Expression Analysis

The reads were quality checked with fastqc [51]. Low quality bases (−q 15) and illumina adaptors were trimmed with fastp (PMID: 30423086). All reads that passed quality control were mapped with an average of 90% uniquely mapped reads to the dog reference genome (Can.Fam3.1) by STAR aligner version 2.6.0 [52] as described in a previous study [17,36]. The read abundance was calculated using HTseq and a gtf file (version 105, downloaded and modified in March 2019) obtained from NCBI Can.Fam3.1 annotation release 105 [53]. The DESeq2 R package [52] in RSTUDIO [54] was used to read the HTseq count data. Pre-filtering was applied for low-expressed/non-expressed genes, excluding those genes where the sum of raw counts across samples was below 10.

The raw counts were subjected to a regularized-logarithm transformation and a principal component analysis (PCA) was performed using R software package “ggplot2” [55] to visualize the clustering of the samples derived from the epidermis and different hair cycle stages. In addition, PCAs, including breed and age, were performed.

To take the gene length into account, raw counts were normalized using the Transcripts Per Million (TPM) method to examine the most expressed genes in each group.

Gene descriptions and functions were identified using the National Center for Biotechnology Information (NCBI) Gene database [56].

### 2.7. Differentially Gene Expression

DEGs between late anagen and telogen samples were analyzed by using a generalized linear model (GLM) of DESeq2 R Package [52] to the pre-filtered raw count data, assuming a negative binomial distribution. Adjusted *p*-values were calculated according to the Benjamini Hochberg method. Transcripts were considered to be differentially expressed with a false discovery rate (FDR) of <0.01 and log2 fold change of 1.5 as the threshold. A heatmap was drawn for the top 30 DEGs sorted based on log2 fold change. The heat map was obtained using DESeq2 normalized gene counts and “heatmap.2” function of “gplots” R package.

### 2.8. Pathway and Gene Enrichment Analysis

Gene Ontology (GO) analysis was applied to analyze the functional enrichment and significant pathways associated with DEGs using the Database for Annotation Visualization and Integrated Discovery (DAVID) [57,58]. The gene symbols of the DEGs were uploaded into the database and the dog genome (*Canis lupus familiaris*) was selected as the background parameter. The upregulated and downregulated DE gene sets comparing late anagen and telogen were used to extract functional gene sets by “functional annotation chart” analyses. Annotations whose Benjaminin corrected *p*-value were lower than 0.05 were considered statistically significant.

## 3. Results

### 3.1. Microdissection

In skin samples from six dogs, either anagen or telogen HF stages predominated. However, in samples from two dogs, both hair cycle stages were present in roughly equal proportions (dogs 4 and 8).

### 3.2. Histologic Findings

Histological examination revealed that, while in most skin samples, both anagen and telogen hair cycle stages were present, one of the stages predominated and corresponded to the stage recognized during microdissection. No pathologic changes were present in the samples included in this study and the epidermis was of normal thickness in all samples.

### 3.3. Gene Expression Profile in Anagen HF, Telogen HF and IFE

Transcriptome sequencing revealed the expression of 21,790 genes (genes are listed in Supplementary Table S2). Principal component analysis (PCA), based on gene expression levels, resulted in the distinct clustering of the different groups (IFE, telogen and late anagen, respectively) (Supplementary Figure S2). However, two of the telogen samples (dogs 2 and 5) clustered distant from the other telogen samples but also from late anagen samples. We therefore concluded that these two samples represent early anagen HFs and not telogen HFs. In fact, these two hair cycle stages cannot be differentiated histologically [49]. These two samples were excluded from the following evaluations in order to compare late anagen and telogen stages only. The influence of age and breed on the differential gene expression was excluded since clustering in PCA plots was not associated with these conditions (Supplementary Figures S3 and S4, respectively). As all samples, except one, were derived from female spayed dogs, the influence of gender could not be evaluated.

### 3.4. Highly Expressed Genes

**Figure 1:**
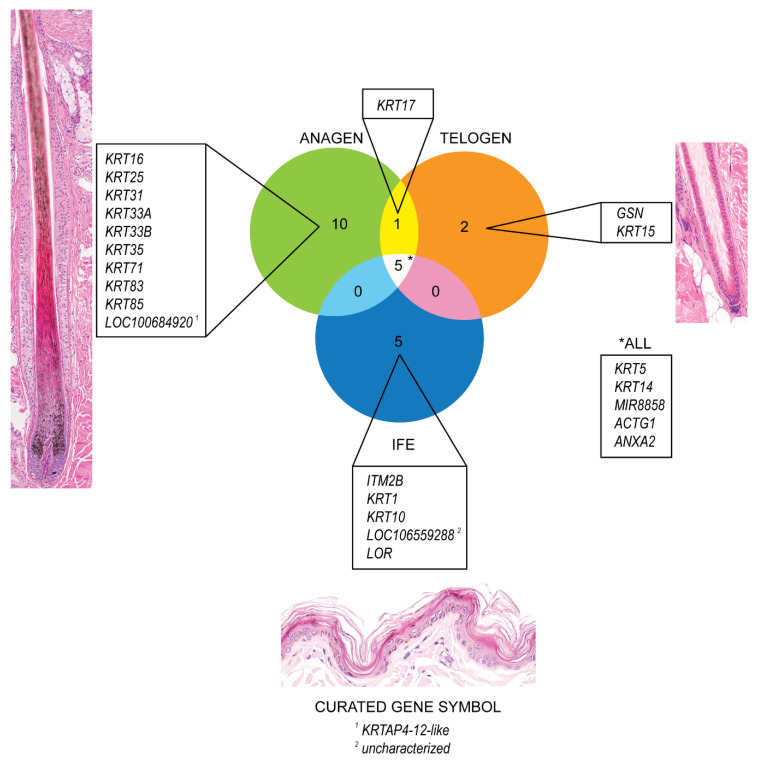
Venn Diagram depicting the most strongly expressed genes (average normalized counts > 5000) in telogen and late anagen canine hair follicles and the interfollicular epidermis, respectively. Genes with the highest expression in each group (late anagen, telogen and interfollicular epidermis) and those which are expressed in more than one group are outlined.

The genes with the highest expression in anagen and telogen HFs and in the IFE were identified and are presented in a Venn diagram using GeneVenn [59] (Figure 1). A high expression level was chosen (average normalized counts > 5000) to single out the most highly expressed genes in the different groups. In late anagen HFs, genes encoding for structural proteins (*KRT16*, *KRT25*, *KRT31*, *KRT33A*, *KRT33B*, *KRT35*, *KRT71*, *KRT83*, *KRT85*, *LOC100684920*) were highly expressed. The LOC gene is predicted to encode keratin-associated protein 4-12-like In telogen HFs, gelsolin (*GSN*) was highly expressed, which encodes a calcium-regulated protein that functions in the assembly and disassembly of actin filaments and therefore plays a role in cellular structure. *KRT15*, a gene encoding for a stem cell marker described to be present in anagen and telogen hair cycle stages in dogs [10], is one of the highest expressed gene in telogen HFs, but also shows a strong expression in late anagen HFs and the IFE, but to a lesser degree (Table 1, Supplementary Table S2).

Most genes that are highly expressed in the IFE are known to be associated with differentiation of human keratinocytes or encode for proteins in the human epidermis (*KRT1*, *KRT10*, *LOR* and *ITM2B*). One of the most expressed genes in the epidermis, LOC106559288, is predicted to encode a long non-coding RNA (lncRNA).

The basal cell markers, *KRT14* and *KRT5*, were strongly expressed in all three groups (IFE, late anagen and telogen), as well as *MIR8858*, *ACTG1* and *ANXA2*, which encode for structural proteins or for proteins associated with cellular growth and signal transduction. 

### 3.5. Gene Expression Profile of Selected Genes

A thorough literature search was performed to search for genes most commonly associated with hair cycle, stem cell markers, HF and IFE structure/function and immune function. Altogether, 131 genes were identified during this search. Of those, 92 genes had an average normalized count of >10 in at least one of the groups (IFE, late anagen, telogen) and were further analyzed. They were assigned to a specific biological function (hair cycle (*n* = 32), stem cell markers (*n* = 12), HF (*n* = 25) and IFE (*n* = 12) structure/function and immune function (*n* = 11), respectively) and used for further analyses. Genes that were expressed at a very low level in all three groups (average normalized count < 10) were not included in further investigations. The average expression of each gene was calculated for IFE, anagen and telogen samples (Table 1).

Genes expressed at least four-fold higher (average normalized count > 100) in one group compared to the others are mentioned in the following. Genes expressed higher in anagen than in the other groups were mainly associated with HF structure (*HOXC13*, *KRT25*, *KRT31*, *KRT33A*, *KRT33B*, *KRT34*, *KRT35*, *KRT71*, *KRT83*, *KRT85*, *KRT86*, *KRTAP16-1*, *PMEL* and *TCHH*). *PMEL* encodes for melanogenic proteins. As melanogenesis takes place in the inferior portion of HFs, which is only present in anagen HFs, it is not surprising that *PMEL* is highly expressed in late anagen HFs. The other genes expressed higher in anagen were associated with the hair cycle (*FOXN1, HSD17B14* and *MSX2*).

In contrast, genes expressed higher in telogen were associated mostly with the hair cycle (*BMP2*, *FGF18*, *MMP7*), but also with stem cell marker (*KRT15*) or follicular structure (*KRT17*).

The selected genes that showed higher expression in the IFE are encoding for epidermal structure (*DSC1*, *KRT1*, *KRT10* and *LOR*). Some of the hair cycle-associated genes (*DKK3* and *WNT3*) are highly expressed in the IFE. Selected genes that expressed strongly in all three groups (average normalized count > 1000 counts) were associated with epidermal and HF structure and function, such as *DSP*, *KRT5*, *KRT14*, *KRT16* and *KRT17,* as well as the stem cell marker *KRT15*.

### 3.6. Expression of Genes Associated with Canine Genodermatoses

The presence and the expression level of genes identified to be associated with canine genodermatoses either affecting the IFE or HFs were analyzed in our data set of normal tissue. All genes known to be associated with canine genodermatoses could be retrieved in our samples. In most cases genes associated with either primarily epidermal or alopecic disease are expressed in both HFs and IFE and it remains to be elucidated why pathology affects only one anatomical structure. The genes that were identified with specific genodermatoses are depicted in Table 2.

### 3.7. Differentially Expressed Genes in Anagen and Telogen Hair Cycle Stages

**Figure 2:**
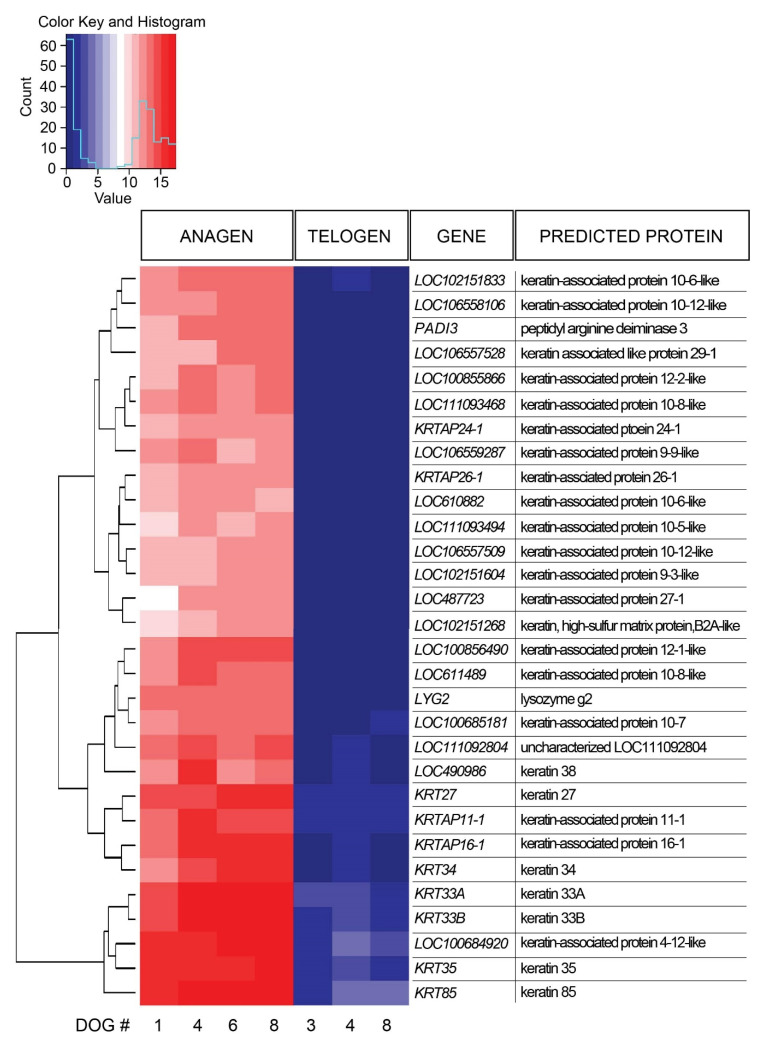
Heat map presenting hierarchical clustering of the 30 most significantly differentially expressed genes comparing late anagen and telogen hair cycle stages. High gene expression is shown in red, low expression in blue, intermediate DESeq2 normalized values in white and different shades of red and blue.

A total of 1236 DEGs were identified in late anagen HFs compared with telogen HF samples, including 858 upregulated genes and 378 downregulated genes (Supplementary Table S3). The 30 most significantly DEGs were further evaluated (Figure 2). All of these genes were expressed significantly more in anagen. Most genes designated with LOC encode keratin-associated proteins, therefore, 90% of the most DEGs encode keratins and keratin-associated proteins. One of the most expressed DEGs (LOC111092804), is uncharacterized and its function is yet unknown.

**Figure 3:**
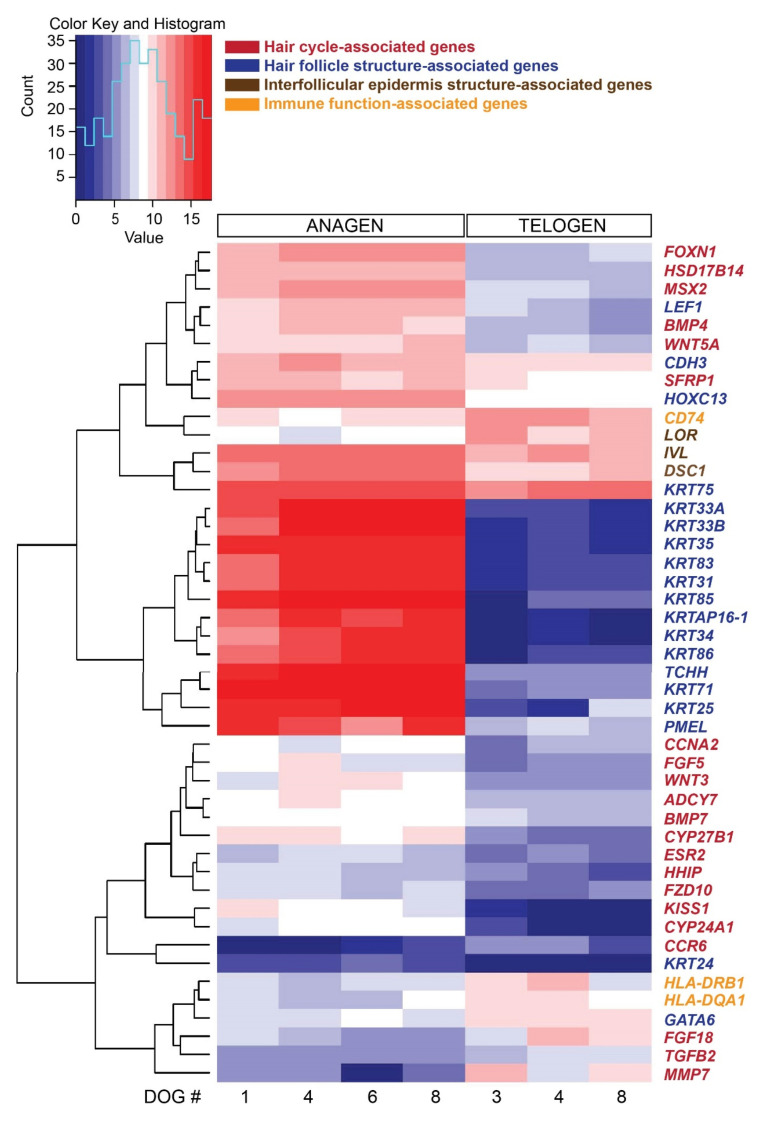
Heat map presenting hierarchical clustering of a preselected subset of 46 genes, based on their known function associated with hair cycle, hair follicle structure and function and stem cells, comparing late anagen and telogen hair cycle stages. High expression is shown in red, low expression in blue, intermediate DESeq2 normalized values in white and different shades of red and blue.

From the 92 preselected genes known to be associated with HF structure and function, hair cycle, stem cells and immune function, 46 genes were differentially expressed comparing late anagen versus telogen hair cycle stages (Figure 3). As presented above, several genes associated with HF structure are expressed mainly in anagen. Genes associated with the hair cycle and expressed significantly more in late anagen are *FOXN1*, *HSD17B14* and *MSX2* and in telogen *FGF18* and *MMP7*. Immune response-associated genes are more strongly expressed in telogen HFs, such as *HLA-DRB1* and *CD74* (both encoding for MHC class II proteins). *KRT75*, *IVL* and *LOR* were strongly expressed by both hair cycle stages, but *KRT75* and *IVL* were expressed significantly more in late anagen and *LOR* in telogen.

### 3.8. Functional Classification of Differentially Expressed Genes

Differentially expressed genes of three GO categories (cellular component, biological process and molecular function) were either up- or down-regulated in anagen. Upregulated and downregulated DEGs enriched GO terms with a Benjamini adjusted *p* value < 0.05 are shown in Supplementary Figure S5. The upregulated genes in anagen are genes related to “keratin filament”, “intermediate filament” and “structural molecule activity”. In contrast, downregulated genes are involved in the “MHC class II protein complex”, “Peptide antigen binding” and “Transmembrane receptor protein tyrosine kinase signaling pathway”.

## 4. Discussion

We studied the gene expression profile of canine microdissected HFs in telogen and late anagen and in the IFE to gain information about the canine-specific expression pattern. It is well known that gene expression in different species varies and our data provide the basis to interpret studies involving gene expression in canine skin disorders, either inherited or acquired, in more detail and with more confidence.

A comparative analysis of the 100 most highly expressed skin-associated genes in human and mouse transcriptomes revealed a 30.2% identity and thus highlights the significant differences between these two species [85]. To investigate similarities/differences in the expression of canine skin-associated genes in relation to humans and mice, we compared the data obtained from our canine samples to the 100 most highly expressed genes in the skin of mice and humans. We found that about 60% of the genes highly expressed in human skin are also strongly expressed in dogs (>100 read counts), whereas only about 45% of the top 100 genes expressed in murine skin were strongly expressed in dogs. Eighteen out of the top 100 skin-associated genes in humans were not expressed in our tissue samples, whereas, compared to the top 100 skin-associated genes in mice, 34 genes were not expressed in our samples (Supplementary Table S4). Of note, the expression data sets derived from human and murine skin were obtained from whole skin and therefore, not surprisingly, contained some highly expressed genes encoding for the mesenchymal components of the skin. Although comparing the expression pattern of whole skin and microdissected tissue is suboptimal, we can conclude that more genes expressed in mice are not expressed in the dog samples as compared to the human skin and gene expression in canine skin is more similar to human skin than in mouse skin. Importantly it has to be mentioned that many of the genes expressed in both, murine and human skin were also strongly expressed (average normalized counts > 100 counts) in at least one of our sample groups (e.g., *DSP*, *KRT1*, *KRT5*, *KRT10*, *KRT14*, *KRT15*, *KRT17* and *LOR*).

These results support that the dog is a good model for human skin disease, as we show in this study that the canine gene expression in IFE and HFs is much more similar to human follicular and epidermal gene expression, as compared to the mouse. Furthermore, our results emphasize the similarity between human and dog signaling associated with hair growth and highlight a more different gene expression pattern in mice. Similarity in the gene expression patterns of dogs and humans, as opposed to mice, can be seen, for example, in WNT expression. In the human anagen HFs, *WNT3* seems to be the most prominent WNT ligand, whereas in the mouse, *Wnt10a* is one of the most prominent WNT ligands [86,87]. Similar to the human anagen HFs, *WNT3* is the most prominently expressed WNT ligand in our anagen samples in dogs.

Examples to underline the difference in the gene expression pattern between dogs and mice is the expression of *KRT17* and *BMP2*. *KRT17* was highly expressed in all our samples (Table 1) but was expressed the most in telogen HFs. This is in contrast to *KRT17* expression in mice, where it is expressed weakly in telogen and expression increases during early anagen and has stable expression in mid- and late anagen [88]. Furthermore, in our samples, *BMP2* was mainly expressed in telogen HFs. This is in contrast to *BMP2* expression in mice, where it is reported to be mainly expressed in late anagen and absent in late telogen [89]. In Cashmere goats, similar to our findings, *BMP2* expression is higher in late telogen and early anagen phases and lower in late anagen [90]. To our knowledge, the specific expression of *BMP2* in different hair cycle stages of human HFs has not yet been described and further studies are warranted to compare human BMP2 expression to that in other species.

We also found differences in the gene expression in the canine IFE and HF samples compared to both mice and human. One example for this is the lack of KRT17 expression in the epidermis of wild type mice and humans [91,92], whereas KRT17 is strongly expressed in canine IFE (Table 1). Another example is the expression of KRT75 in both anagen and telogen HFs in dogs, whereas *KRT75* is constitutively expressed only in the anagen-stage HFs in mice and humans [93].

Gene expression profiling in canine microdissected HFs, comparing late anagen and telogen hair cycle stages, revealed a significantly higher expression of keratins and keratin-associated proteins, as well as of two other genes, namely *PADI3* and *LYG2,* in anagen HFs. *PADI3* modulates hair structural proteins, such as filaggrin and trichohyalin in the inner root sheath during HF formation, and is therefore equally associated with HF structure and function. *LYG2* was enriched in the WNT signaling pathway in a study investigating DEGs in the skin of Liaoning cashmere goats [43]. The function of this gene in canine HFs needs still to be determined. The genes expressed strongest in telogen HFs are *GSN* and *KRT15*. However, both of these genes are also strongly expressed in the other tissue groups (Supplementary Table S2).

In the IFE, the genes that are expressed highest are associated with keratinocyte differentiation. One of the most expressed genes in canine IFE, *LOC106559288*, encodes a lncRNA, which is transcribed from the keratin gene cluster on dog chromosome 9. Further investigation is warranted whether this predicted lncRNA represents an artifact of the imperfect canine genome assembly and annotation or whether it has a real biological function. Genes expressed highly in all three tissue groups are involved in cellular structure, the regulation of cellular growth and signal transduction (*KRT5*, *KRT14*, *ACTB*, *ANXA2*, *MIR8858*).

Some of the hair cycle-associated genes (*DKK3* and *WNT3*) are also highly expressed in the IFE, similar to mice and humans [94,95]. The role of these two genes in epidermal homeostasis is not yet clear but there is evidence that WNT/β-catenin signaling is involved in the maintenance of the epidermal stem cell pool [94]. *DKK3*, in contrast to other members of the Dickkopf family, does not interfere with the WNT signaling pathway and is proposed to be a tumor suppressor gene.

Analyzing genes in our data sets known to be associated with HF and IFE structure and function, stem cell markers and immune function, we found that, in anagen, the hair cycle-associated genes that are most upregulated are *MSX2*, *FOXN1* and *HSD17B14*, and the most significantly differently expressed genes associated with HF structure (apart from keratins) is *HOXC13.* It has been suggested previously that *FOXN1* and *HOXC13* are required for hair keratin expression in mice and humans [96,97] and *HOXC13* was also shown to be highly expressed in human anagen HFs [97]. *FOXN1* (formerly called *whn* and *hfh11*) is known to be predominantly expressed in hair matrix keratinocytes, the hair cortex, and in outer root sheath keratinocytes and is known, together with *Msx-2,* to maintain anagen in mice [98,99]. The high expression of HOXC13, FOXN1 and MSX2 in canine HFs may suggest that these genes have a similar function, as compared to humans and mice.

In telogen, the most significantly DEGs associated with the hair cycle were *FGF18* and *MMP7*. *FGF18* is an important indicator of stem cell quiescence. In mice, *Fgf18* was found to be expressed in the bulge and *FGF18* peaked during telogen [100]. The deletion of *Fgf18* in mice results in a shorter telogen phase [22,101]. Our results are in line with this and also support that, in dogs, *FGF18* is a telogen marker. While not much is known about the expression of matrix metalloproteinase 7 (*MMP7*) in HFs, *Mmp2* and *Mmp9* expression was shown in all structures of murine HFs and in the sebaceous glands of different hair cycle stages, respectively [102]. In our canine study, *MMP7* seems to be predominantly expressed in telogen HFs, suggesting a role in the hair cycle of dogs.

Surprisingly, most of the selected stem cell-associated genes did not show significantly different expression between anagen and telogen. These results are similar to results from a recent study comparing anagen and telogen HFs in Cashmere goats. In this study, only *LHX2* and *NFATC1* were differentially expressed [42]. In our study, *NFATC1* was expressed in both late anagen and telogen, whereas *LHX2* was expressed strongly in telogen only (Table 1). Since stem cell activity is highest in early anagen follicles, we assume that the comparison of late anagen and telogen is not optimal to detect differences in stem cell activity.

All genes playing a role in the immune function of the HFs, including β2 microglobulin *(B2M)* and canine MHC class I molecules (*DLA88*, *DLA-79*, *DLA-12*, *DLA-64*) were expressed at very low levels in late anagen HFs and much higher in telogen HFs and the IFE (Supplementary Table S2). This is in line with the knowledge that the inferior portion of the anagen HF is one of the immune-privileged sites of the body. Immune-privilege is achieved by, for example, the low expression of MHC class I molecules and *B2M* [103] and shields the HFs from autoimmune attacks, as happens in alopecia areata [104,105], a common disease in humans and domestic animals. Our data thus strongly indicate, that, like in humans, the inferior portion of the anagen HF is an immune-privileged site and identify the molecules associated with this privilege. In addition, our results indicate that dogs have a similar gene expression pattern associated with follicular immunoregulation in late anagen and, thus, the dog may be a suitable model to study the pathogenesis of alopecia areata.

Comparing our datasets with genes that have been associated with inherited canine epidermal disease and alopecia in the past, we identified the expression of all of these genes, with the exception of RSPO in the IFE, in all three tissue groups. Why genetic variants result in pathology only in the epidermis needs to be further investigated. Generally speaking, the genes identified in conjunction with alopecia are expressed at much lower levels. Interestingly, even though ichthyosis usually exclusively affects the epidermis, one of the genes associated with ichthyosis occurring in Great Danes (*FATP4* [71]) had a higher expression in the anagen HF group compared to the IFE samples in our data set. This form of ichthyosis is the only one described in the literature that also has pathologic lesions in the folliculosebaceous units [106], which could be explained by the expression of *FATP4* in anagen HFs in our data.

## 5. Conclusions

We presented the gene expression pattern of canine IFE and microdissected HFs in different hair cycle stages (late anagen and telogen). We found that the gene expression patterns of skin-associated genes in dogs are more similar to those in humans as compared to mice, further corroborating that the dog is a good model for human skin diseases. We also found gene expression that differs from what is known in mice and humans, such as *BMP2* expression mainly in telogen in dogs but not mice, and *KRT17* expression in the IFE in dogs, while absent in mice and humans. Our data provide the basis for research investigating the structure and function of canine skin or skin disease, but also for scientists using a canine model for human disease, by assigning genes to specific tissue types. 

## Figures and Tables

**Table 1:** Expression levels of 92 selected genes. Preselected hair cycle-associated, hair follicle structure-associated, stem cell-associated, immune function-associated and epidermal structure-associated genes are listed, and the expression levels based on the average TPM normalized values in telogen, anagen and interfollicular epidermis samples are depicted. Darker colors represent higher expression levels (average normalized counts ≥ 400), lighter shades of blue represent intermediate and low expression levels (average normalized count of ≥100 and <400 and ≥10 and <100, respectively). White boxes represent very low expression of genes (average normalized counts < 10).

**Hair Cycle-Associated Genes**				
**Gene Symbol**	**Full Name of Gene**	**Anagen**	**Telogen**	**IFE**
*ADCY7*	Activin A receptor type 1	18.64	3.77	5.48
*BMP2*	Bone morphogenetic protein 2	44.37	281.79	66.23
*BMP4*	Bone morphogenetic protein 4	99.16	7.29	12.64
*BMP7*	Bone morphogenetic protein 7	25.65	7.97	22.11
*BNC1*	Basonuclin 1	70.47	59.68	83.80
*BNC2*	Basonuclin 2	48.34	40.38	4.74
*CCNA2*	Cyclin A2	29.04	6.11	9.87
*CYP1A1*	Cytochrome P450 family 1 subfamily A member 1	17.39	43.18	74.82
*CYP1B1*	Cytochrome P450 family 1 subfamily B member 1	96.94	214.59	129.88
*CYP24A1*	Cytochrome P450 family 24 subfamily A member 1	18.89	0.12	0.00
*CYP27B1*	Cytochrome P450 family 27 subfamily B member 1	57.64	1.45	0.96
*DKK3*	Dickkopf 3	102.28	129.90	218.67
*FGF18*	Fibroblast growth factor 18	8.70	133.54	4.28
*FGF5*	Fibroblast growth factor 5	53.66	4.65	1.78
*FOXN1*	Forkhead box protein N1	182.11	5.58	24.39
*FZD10*	Frizzled 10	15.66	3.34	3.48
*FZD3*	Frizzled 3	16.46	9.32	2.64
*HSD17B14*	Hydroxysteroid 17-beta dehydrogenase 14	396.58	17.20	25.22
*JAG1*	Jagged 1	30.05	22.36	13.30
*KISS1*	KISS-1 metastasis-suppressor	60.79	0.17	0.00
*MMP7*	Matrix metalloproteinase 7	5.44	218.73	12.82
*MSX2*	Homeobox protein MSX2	771.09	49.38	64.14
*SFRP1*	Secreted frizzled related protein 1	107.40	37.31	18.18
*SFRP2*	Secreted frizzled related protein 2	39.53	14.07	0.26
*SOX10*	Sex determining region Y-box 10	54.93	59.27	12.94
*TCF7/TCF1*	Transcription factor 1	55.84	46.93	4.60
*TGFB2*	Transforming growth factor beta 2	2.53	11.32	5.62
*THBS2*	Thrombospondin 2	67.86	137.49	62.01
*VDR*	Vitamin D receptor	105.58	55.58	22.71
*WNT3*	Wnt family member 3	22.82	1.93	97.02
*WNT5a*	Wnt family member 5A	36.11	5.15	12.76
*WNT10A*	Wnt family member 10A	27.32	18.12	20.25
**Hair Follicle Structure-Associated Genes**				
**Gene Symbol**	**Full Name of Gene**	**Anagen**	**Telogen**	**IFE**
*CD133/PROM1*	Prominin	15.86	3.44	0.86
*CD71/TFRC*	Transferrin receptor	61.61	31.88	23.85
*CDH3*	P-Cadherin 3	196.09	225.99	463.31
*GATA6*	GATA binding protein 6	14.80	59.76	4.07
*HOXC13*	Homeobox C3	374.22	41.39	13.79
*KRT16*	Keratin 16	5931.82	10,187.55	1790.56
*KRT17*	Keratin 17	15,476.37	77,099.96	8158.26
*KRT25*	Keratin 25	10,456.27	33.58	2.57
*KRT31*	Keratin 31	5422.80	2.05	1.89
*KRT33A*	Keratin 33A	10,416.91	2.24	7.39
*KRT33B*	Keratin 33B	10,528.67	1.77	2.25
*KRT34*	Keratin 34	3966.39	0.55	0.81
*KRT35*	Keratin 35	5365.72	1.11	0.36
*KRT6A*	Keratin 6A	1018.09	1513.19	37.94
*KRT6B*	Keratin 6B	3692.26	8621.62	80.91
*KRT71*	Keratin 71	17,621.04	11.18	7.17
*KRT75*	Keratin 5	2585.70	2895.72	2.59
*KRT79*	Keratin 79	279.52	3752.50	1216.86
*KRT83*	Keratin 83	5671.73	2.30	2.07
*KRT85*	Keratin 85	10,133.20	2.73	2.81
*KRT86*	Keratin 86	3068.60	1.20	1.06
*KRTAP16-1*	Keratin associated protein 16-1	2984.34	0.33	0.72
*LEF1*	Lymphoid enhancer binding factor 1	87.27	18.84	21.65
*PMEL*	Premelanosome protein	1907.90	21.59	135.56
*TCHH*	Trichohyalin	3997.42	5.24	8.35
**Stem Cell-Associated Genes**				
**Gene Symbol**	**Full Name of Gene**	**Anagen**	**Telogen**	**IFE**
*CD200*	CD200 Molecule	23.69	33.44	6.71
*CD34*	CD34 Molecule	111.79	131.55	47.56
*ITGA6*	Integrin subunit alpha 6	70.85	189.30	150.07
*ITGB1*	Integrin subunit beta 1	219.44	341.60	265.09
*KRT15*	Keratin 15	2485.12	17,673.04	4104.93
*KRT19*	Keratin 19	59.14	180.62	4.04
*LGR5*	Leucin rich repeat containing G protein-coupled receptor 5	9.43	10.24	0.02
*LHX2*	LIM homeobox 2	105.49	200.13	0.34
*NFATC1*	Nuclear factor of activated T cells	19.16	76.54	21.90
*SOX9*	SRY-box transcription factor 9	563.03	817.93	104.80
*TCF3*	Transcription factor 3	127.36	105.95	82.50
*TCF4*	Transcription factor 4	42.90	104.53	40.37
**Epidermal Structure-Associated Genes**				
**Gene Symbol**	**Full Name of Gene**	**Anagen**	**Telogen**	**IFE**
*DSC1*	Desmocollin 1	328.67	71.79	1523.99
*DSG1*	Desmoglein 1	505.83	479.42	984.35
*DSP*	Desmoplakin	1406.58	1492.09	1610.92
*FLG*	Filaggrin	12.35	71.70	60.74
*IVL*	Involucrin	2244.92	480.55	85.91
*KRT1*	Keratin 1	203.64	988.44	21,712.07
*KRT5*	Keratin 5	13,982.27	18,257.94	12,733.06
*KRT10*	Keratin 10	339.13	2042.75	33,847.68
*KRT14*	Keratin 14	23,040.65	19,320.49	29,472.32
*LOR*	Loricrin	65.36	399.38	7229.10
*PLEC*	Plectin	55.78	87.56	95.43
*TP63*	Tumor protein p63	137.68	179.75	230.67
**Immune Function-Associated Genes**				
**Gene Symbol**	**Full Name of Gene**	**Anagen**	**Telogen**	**IFE**
*CCL20*	C-C motif chemokine ligand 20	0.78	3.94	14.63
*CD74*	CD74 molecule	113.38	737.23	1498.77
*DLA88*	MHC class I DLA-88	116.10	439.94	584.87
*DLA-DQA1*	MHC class II DQ alpha 1	20.24	115.09	257.38
*HLA-DRB1*	MHC class II DR beta 1	34.90	211.64	455.74
*IL17B*	Interleukin 17B	15.01	11.49	4.35
*IL18*	Interleukin 18	18.55	62.51	94.44
*IL1A*	Interleukin 1 alpha	19.31	6.59	13.99
*IL33*	Interleukin 33	10.40	28.91	17.75
*TLR2*	Toll like receptor 2	1.79	15.28	2.43
*TLR4*	Toll like receptor 4	7.55	17.14	12.41

**Table 2:** Expression of genes associated with canine genodermatoses. Genes that are associated with a genetic variant leading to epidermal or follicular disease or phenotype are listed and the expression levels based on the average TPM normalized values in telogen, anagen and interfollicular epidermis samples are depicted.

Tissue Affected	Disease	Dog Breed	Gene Mutation	Anagen	Telogen	IFE	Ref.
IFE	CHILD’s like syndrome	Labrador retriever, Chihuahua	*NSDHL*	41.2	36.5	21.2	[60,61]
	Darier disease	Irish Terrier	*ATP2A2*	14.3	19.3	18.9	[62]
	Epidermolysis bullosa, dystrophic	Golden retriever	*COL7A1*	9.6	58.6	61.4	[63,64]
	Epidermolysis bullosa simplex, junctional	German shorthaired pointer	*LAMA3*	20.6	58.6	41.4	[65]
	Epidermolysis bullosa simplex, suprabasal	Chesapeake Bay retriever	*PKP1*	0.1	0.2	0.3	[66]
	Epidermolysis bullosa simplex, basal	Eurasier	*PLEC*	55.8	87.6	95.4	[67]
	Hereditary nasal parakeratosis	Labrador retriever, greyhound	*SUV39H2*	9.8	13.9	12.5	[34,68,69]
	Hereditary footpad hyperkeratosis	Irish terrier, Kromfohrländer	*FAM83G*	174.2	190.6	158.6	[35]
	Ichthyosis	German shepherd dog	*ASPRV1*	252.9	111.1	698.8	[70]
		Great Dane	*SLC27A4 (FATP4)*	178.9	125.9	147.6	[71]
		Norfolk terrier	*KRT10*	339.1	2042.8	33,847.7	[72]
		American bulldog	*NIPAL4*	60.9	68.2	250.1	[73,74,75]
		Golden retriever	*PNPLA1*	58.0	46.8	90.3	[76]
		Jack Russell terrier	*TGM1*	83.1	34.2	138.0	[77]
HF	Canine ectodermal dysplasia	Chinese crested, Mexican hairless dogs, Peruvian hairless dog	*FOXI3*	0.4	7.9	0.1	[78]
		German shepherd dog, dachshund	*EDA*	2.4	7.8	4.8	[79,80,81]
		Chesapeake Bay retriever	*PKP1*	0.1	0.2	0.3	[66]
	Hairlessness	American terrier, Scottish deerhound	*SGK3*	74.1	38.1	47.9	[82,83]
	Improper coat	Portuguese water dog	*RSPO*	1.2	0.6	0.0	[84]

## Supplementary Materials

The following are available online at https://www.mdpi.com/2073-4425/11/8/884/s1, Table S1. Dogs used as tissue donors for the experiments. Dogs used as skin tissue donors for the experiments, including breed, sex and age, are listed. Table S2. Overall gene expression. Normalized read counts of all genes expressed in the different hair follicle stages and the epidermis. Gene expression values of individual dogs and average values for hair follicles in late anagen, early anagen, telogen and interfollicular epidermis sample groups are shown. Table S3. Results of differential expression analysis. Significantly differentially expressed genes comparing the base mean of normalized read counts of microdissected late anagen and telogen hair follicles with a false discovery rate (FDR) < 0.01 and log2 fold change of 1.5 as threshold, sorted alphabetically; BaseMean: mean of normalized read counts across all samples; log2 fold change: condition anagen versus telogen effect; LfcSE: standard error of the log2FoldChange; stat: the log2FoldChange divided by lfcSE; pvalue: Wald test effect; padj: Benjamini Hochberg adjusted *p*-values; FDR refers to Benjamini Hochberg adjusted *p*-values. Table S4. Skin-associated genes in humans and mice not expressed in dog skin. The top 100 human and mouse skin-specific genes [59] were compared to expression of these genes in dog skin. Genes highly expressed in human and mouse but not dog skin are listed. Figure S1. Microdissected hair follicles in different hair cycle stages. Hair follicles of both hair cycle stages were cut above the sebaceous glands to clearly separate hair follicles from interfollicular epidermis. A: Late anagen hair follicle. Note that the hair shaft emerges through the hair shaft ostia. B: Telogen hair follicle. Figure S2. Principal component analysis (PCA) in the two-dimensional space of the transcriptome samples. Samples are plotted across the most variable components (PC1 and PC2) and sample clustering is based on late anagen, telogen and interfollicular epidermis. Whereas samples of the late anagen and the interfollicular epidermis group cluster nicely together two telogen samples are clustering separately from the other telogen samples. This leads to the conclusion that these samples represent rather an early anagen stage. Figure S3. Principal component analysis (PCA) in the two-dimensional space of the transcriptome samples. Samples are plotted across the most variable components (PC1 and PC2) and sample clustering is based on age. Figure S4. Principal component analysis (PCA) in the two-dimensional space of the transcriptome samples. Samples are plotted across the most variable components (PC1 and PC2) and sample clustering is based on breed. Figure S5. Functional and pathway enrichment analysis of differentially expressed genes comparing late anagen and telogen samples. Gene Ontology enrichment analysis of differentially expressed genes has been performed using the Database for Annotation Visualization and Integrated Discovery. Enriched pathways and gene ontology terms that are upregulated or downregulated in anagen hair follicles are listed and their biological function is indicated in different colors.
