# Supplementary material for: Transcriptome Profiling and Differential Gene Expression in Canine Microdissected Anagen and Telogen Hair Follicles and Interfollicular Epidermis
Source: Genes (Basel). 2020 Aug 4;11(8):884. doi: 10.3390/genes11080884 (PMC7463739; doi:10.3390/genes11080884)
Supplement: Supplementary file 1 [file genes-11-00884-s001.zip › S1 Table.docx]

**S1 Table. Dogs used as tissue donors for the experiments.** Dogs used as skin tissue donors for the experiments, including breed, sex and age, are listed.

| Dog number | Breed | Age | Gender | Tissue for RNAseq |
| --- | --- | --- | --- | --- |
| 1 | Labrador retriever | 10 years | fs | epidermis, hair follicles (anagen) |
| 2 | Labrador mix | 14 years | fs | epidermis, hair follicles (telogen) |
| 3 | Mixed breed | 11 years | fs | epidermis, hair follicles (telogen) |
| 4 | Australian shepherd | 10 years | fs | epidermis, hair follicles (anagen + telogen) |
| 5 | Mixed breed | 9 years | fs | epidermis, hair follicles (telogen) |
| 6 | Great Dane | 4 years | fs | epidermis, hair follicles (anagen) |
| 7 | Chihuahua | 7 months | m | epidermis |
| 8 | Labrador retriever | 15 years | fs | hair follicles (anagen + telogen) |
