# Supplementary material for: Transcriptome Profiling and Differential Gene Expression in Canine Microdissected Anagen and Telogen Hair Follicles and Interfollicular Epidermis
Source: Genes (Basel). 2020 Aug 4;11(8):884. doi: 10.3390/genes11080884 (PMC7463739; doi:10.3390/genes11080884)
Supplement: Supplementary file 1 [file genes-11-00884-s001.zip › S4 Table.docx]

**S4 Table. Skin-associated genes in humans and mice not expressed in dog skin.** The top 100 human and mouse skin-specific genes^1^ were compared to expression of these genes in dog skin. Genes highly expressed in human and mouse but not dog skin are listed.

**Skin-specific genes highly expressed in human but not dog skin**

| **GENE NAME** | **GENE SYMBOL** |
| --- | --- |
| Annexin A8 | *ANXA8* |
| Arylacetamide deacetylase-like 2 | *AADACL2* |
| Chromosome 19 open reading frame 33 | *C19orf33* |
| Chromosome 1 open reading frame 68 | *C1orf68* |
| Chromosome 5 open reading frame 46 | *C5orf46* |
| Cysteine-rich C-terminal1 | *CRCT1* |
| Dermcidin | *DCD* |
| Filaggrin family member 2 | *FLG2* |
| G protein-coupled receptor 115 | *GPR115* |
| Keratin-associated protein 2-2 | *KRAP2-2* |
| Late cornified envelop 1B | *LCE1B* |
| Late cornified envelope 2B | *LCE2B* |
| Lymphocyte antigen 6 complex, locus D | *LY6D* |
| Mucin-like 1 | *MUCL1* |
| Secretoglobin, family 1D, member 2 | *SCGB1D2* |
| Secretoglobin, family 2A, member 2 | *SCGB2A2* |
| Serine (or cysteine) proteinase inhibitor, clade A (alpha-1 antiproteinase, antitrypsin), member 12 | *SERPINA12* |
| Zinc finger protein 750 | *ZNF750* |

**Skin-specific genes highly expressed in mouse but not dog skin**

| **GENE NAME** | **GENE SYMBOL** |
| --- | --- |
| RIKEN cdna 2310050C09 gene | *2310050C09Rik* |
| Arachidonate lipoxygenase, epidermal | *Alox12e* |
| Annexin A8 | *ANXA8* |
| Mouse chemokine (C-C motif) ligand 8 | *Ccl8* |
| Chitinase 1 (chitotriosidase) | *Chit1* |
| Cysteine-rich C-terminal 1 | *Crct1* |
| Defensin beta 6 | *Defb6* |
| Desmoglein 1 alpha | *Dsgl1a* |
| Eosinophil-associated ribonuclease 5 | *Ear5* |
| Family with sequence similarity 25, member C | *Fam25c* |
| Filaggrin family member 2 | *Flg2* |
| Growth differentiation factor 8 | *Gdf8* |
| Keratin-associated protein 16-2 | *Krtap 16-2* |
| Keratin-associated protein 28-10 | *Krtap 28-10* |
| Keratin-associated protein 8-2 | *Krtap 8-2* |
| Keratin-associated protein 15 | *Krtap15* |
| Keratin-associated protein 16-4 | *Krtap16-4* |
| Keratin-associated protein 16-5 | *Krtap16-5* |
| Keratin-associated protein 16-8 | *Krtap16-8* |
| Keratin-associated protein 19-9b | *Krtap19-9b* |
| Keratin-associated protein 6-1 | *Krtap6-1* |
| Late cornified envelope 1L | *Lce1l* |
| Major urinary protein 1 | *Mup1* |
| Myosin, heavy polypeptide 4, skeletal muscle | *Myh4* |
| Prosaposin-like 1 | *Psapl1* |
| Stearoyl-coenzyme A desaturase 3 | *Scd3* |
| Short chain dehydrogenase/reductase family 16C, member 6 | *Sdr16c6* |
| Selection and upkeep of intraepithelial T cells 10 | *Skint10* |
| Selection and upkeep of intraepithelial T cells 11 | *Skint11* |
| Selection and upkeep of intraepithelial T cells 4 | *Skint4* |
| Selection and upkeep of intraepithelial T cells 9 | *Skint9* |
| Small protein rich-like 10 | *Sprrl10* |
| Small proline rich-like 2 | *Sprrl2* |
| Small proline rich-like 3 | *Sprrl3* |
