# Supplementary figures and images for: Transcriptome Profiling and Differential Gene Expression in Canine Microdissected Anagen and Telogen Hair Follicles and Interfollicular Epidermis
Source: Genes (Basel). 2020 Aug 4;11(8):884. doi: 10.3390/genes11080884 (PMC7463739; doi:10.3390/genes11080884)

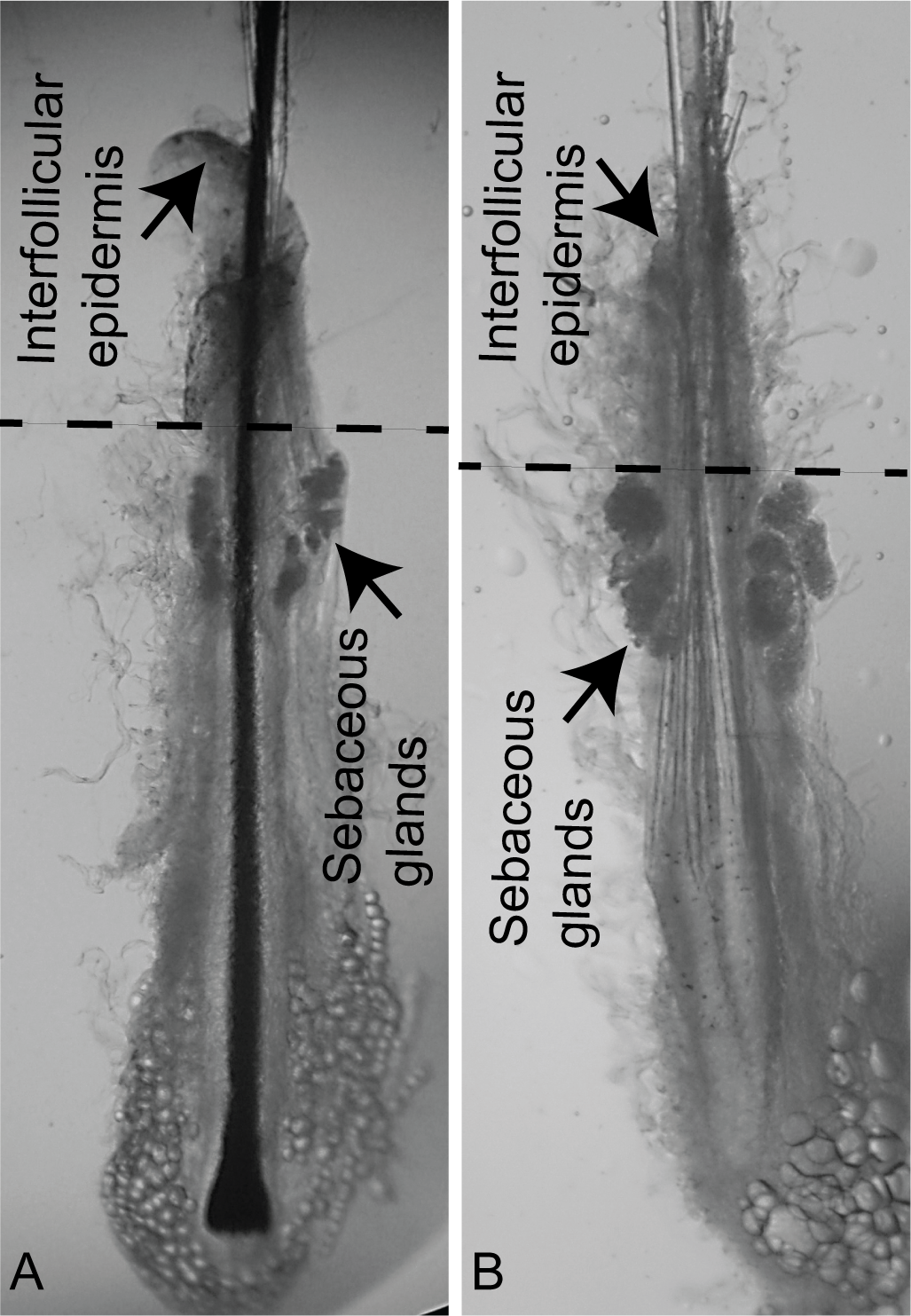

Supplement: Supplementary file 1 [file genes-11-00884-s001.zip › S1 Figure.tif]

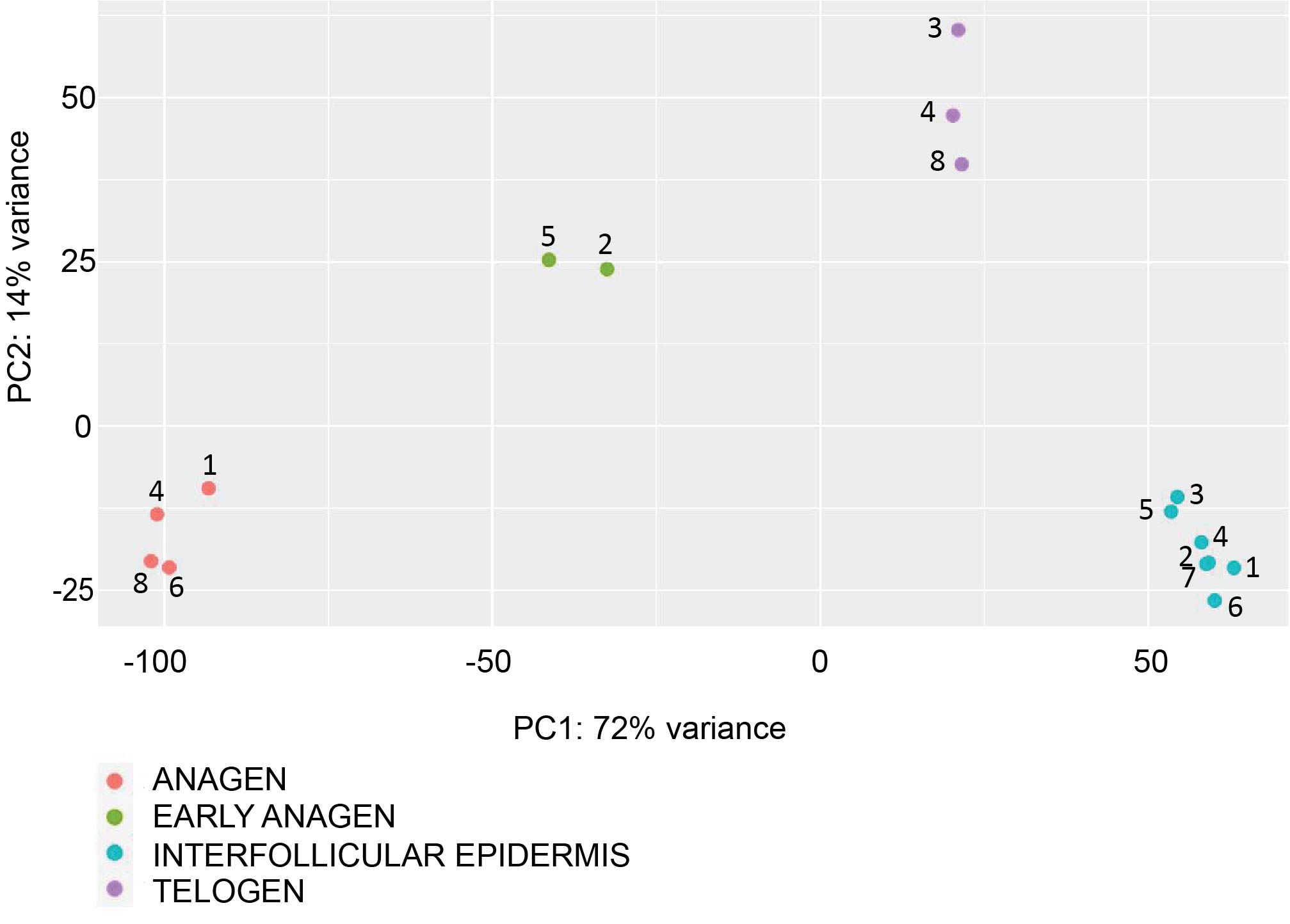

Supplement: Supplementary file 1 [file genes-11-00884-s001.zip › S2 Figure new.jpg]

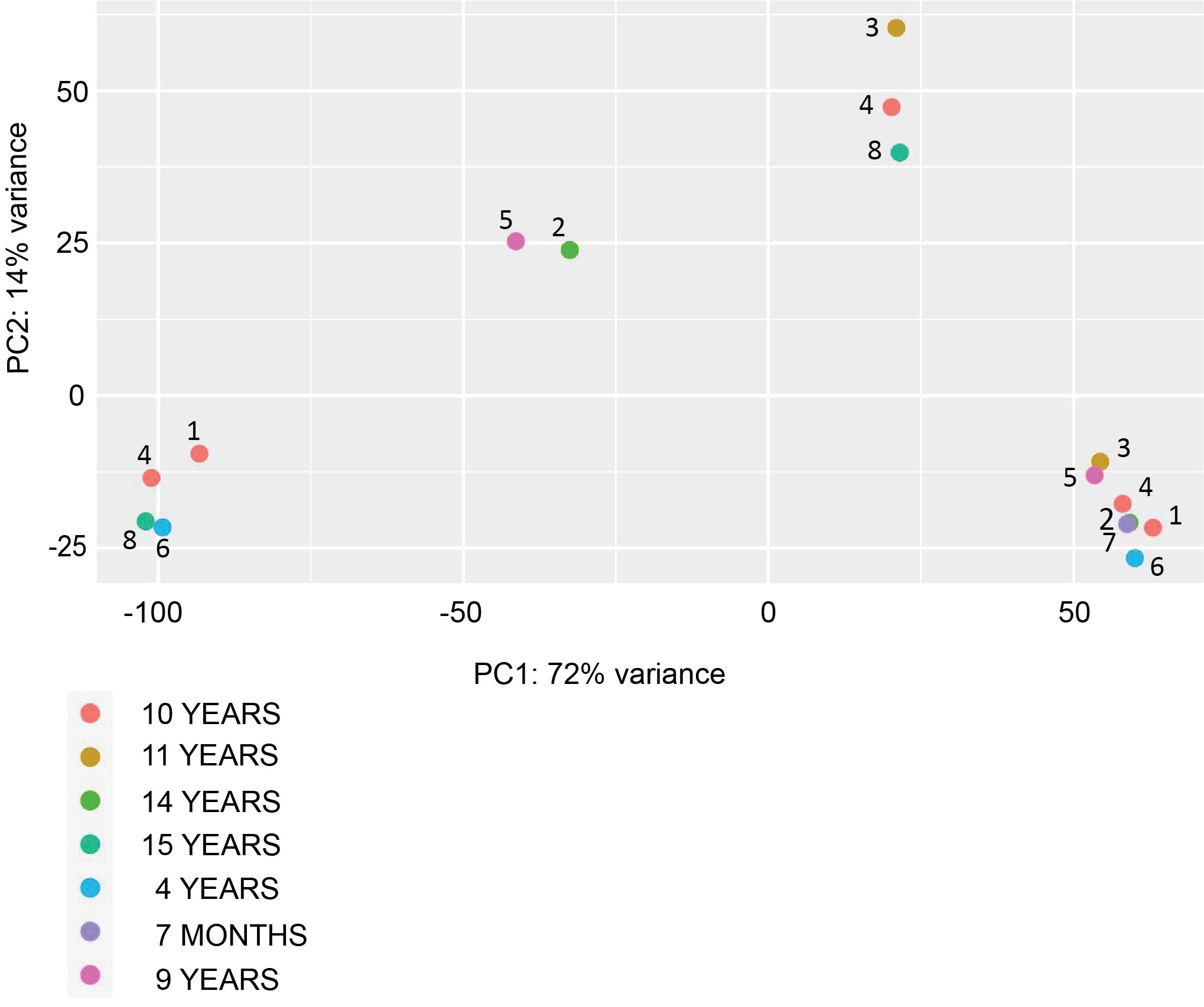

Supplement: Supplementary file 1 [file genes-11-00884-s001.zip › S3 Figure new.jpg]

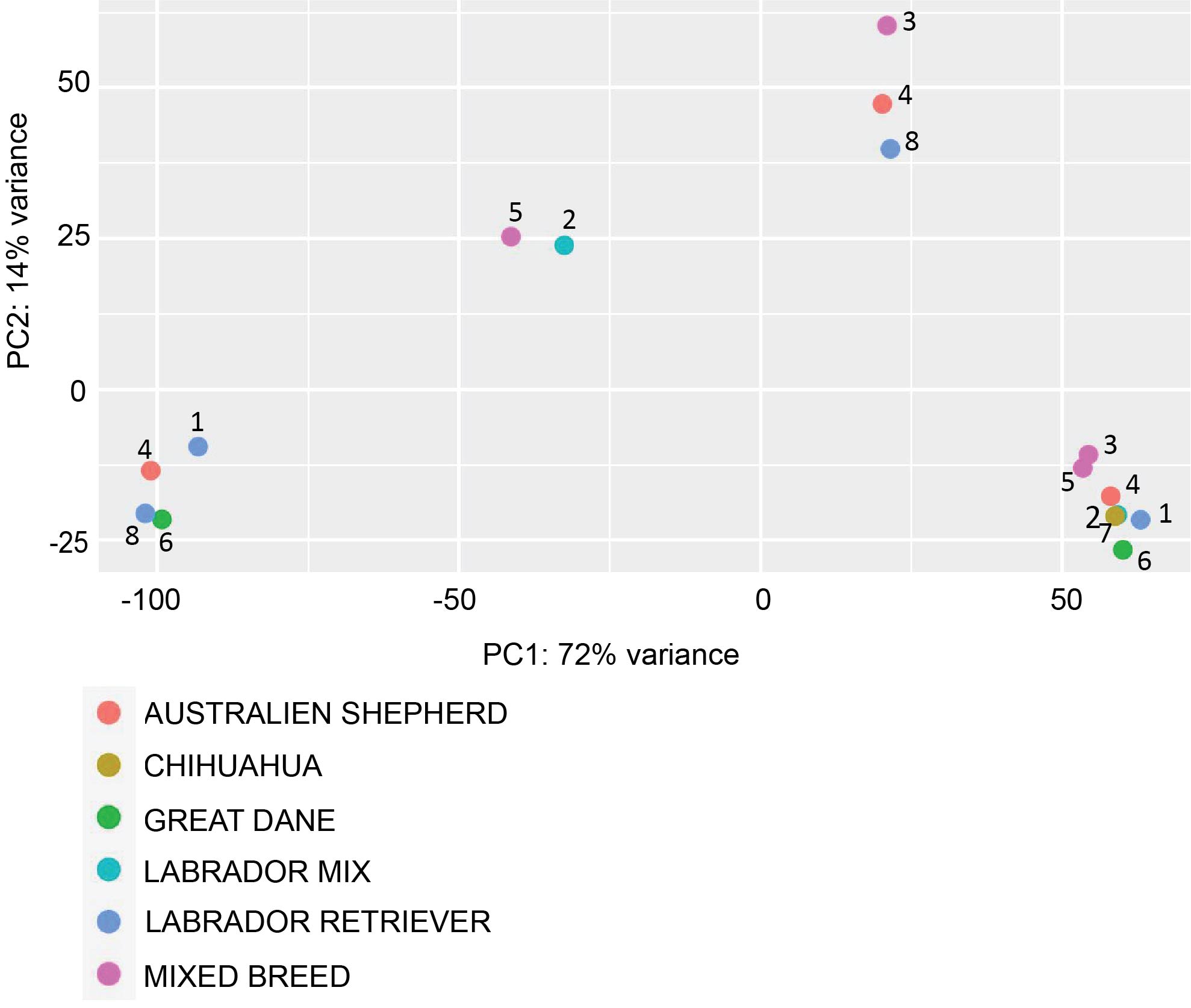

Supplement: Supplementary file 1 [file genes-11-00884-s001.zip › S4 Figure new.jpg]

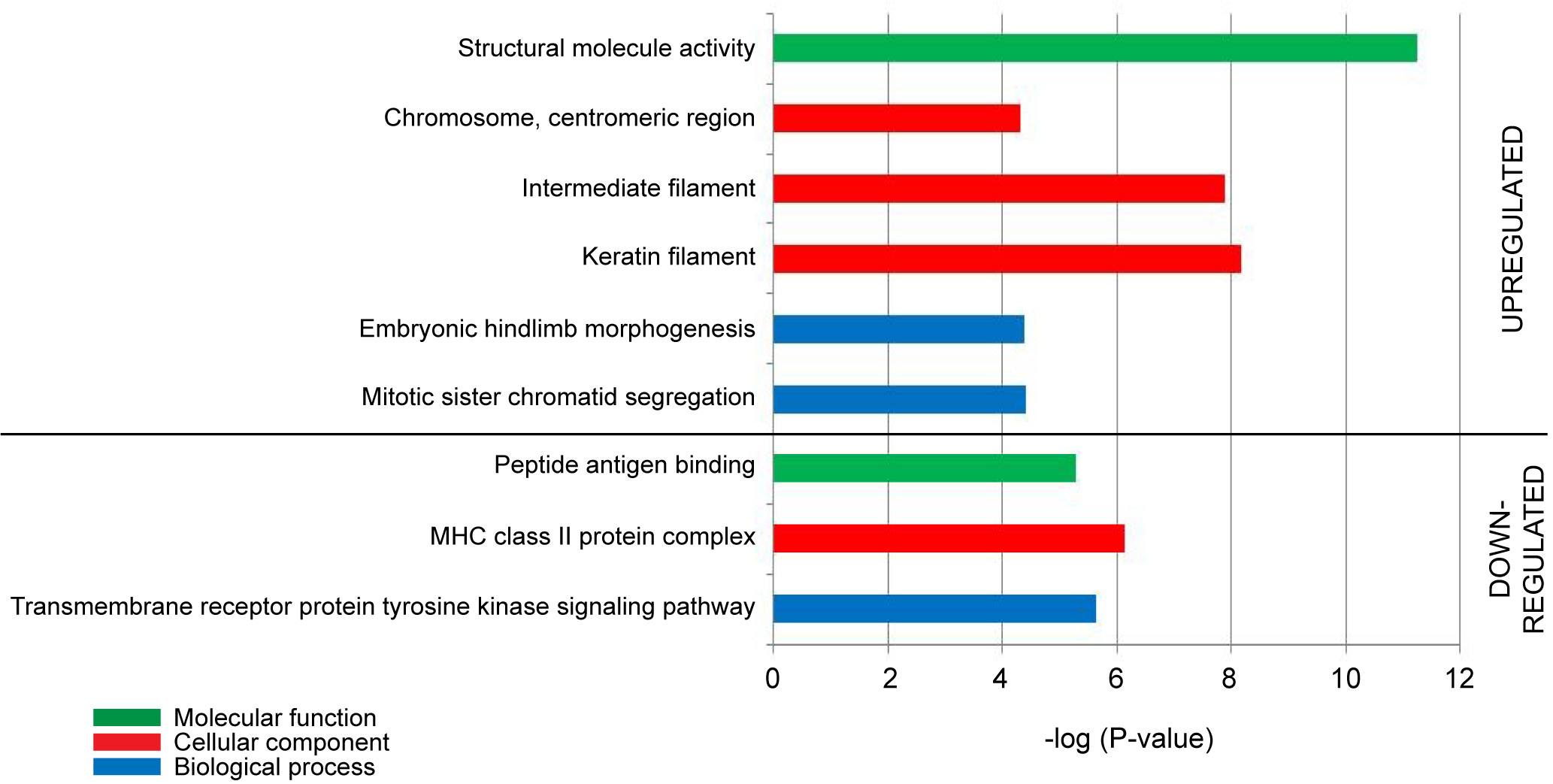

Supplement: Supplementary file 1 [file genes-11-00884-s001.zip › S5 Figure.tif]
